# Impact of illicit opioid use on T cell subsets among HIV-infected adults

**DOI:** 10.1371/journal.pone.0176617

**Published:** 2017-05-04

**Authors:** E. Jennifer Edelman, Kaku So-Armah, Debbie M. Cheng, Margaret F. Doyle, Sharon M. Coleman, Carly Bridden, Natalia Gnatienko, Dmitry A. Lioznov, Elena Blokhina, Matthew S. Freiberg, Evgeny M. Krupitsky, Brinda Emu, Jeffrey H. Samet

**Affiliations:** 1 Department of Medicine, Section of General Internal Medicine, Yale University School of Medicine, New Haven, CT, United States of America; 2 Center for Interdisciplinary Research on AIDS, Yale University School of Public Health, New Haven, CT, United States of America; 3 Department of Medicine, Section of General Internal Medicine, Clinical Addiction Research and Education Unit, Boston Medical Center, Boston, MA, United States of America; 4 Department of Biostatistics, Boston University School of Public Health, Boston, MA, United States of America; 5 Department of Pathology and Laboratory Medicine, University of Vermont, Colchester, VT, United States of America; 6 Data Coordinating Center, Boston University School of Public Health, Boston, MA, United States of America; 7 First Pavlov State Medical University of St. Petersburg, St. Petersburg, Russian Federation; 8 Vanderbilt University, Nashville, TN, United States of America; 9 Nashville Veterans Affairs Medical Center, Nashville, TN, United States of America; 10 St.-Petersburg Bekhterev Research Psychoneurological Institute, St. Petersburg, Russian Federation; 11 Department of Medicine, Section of Infectious Diseases, Yale University School of Medicine, New Haven, CT, United States of America; 12 Department of Medicine, Section of General Internal Medicine, Clinical Addiction Research and Education Unit, Boston University School of Medicine/Boston Medical Center, Boston, MA, United States of America; 13 Department of Community Health Sciences, Boston University School of Public Health, Boston, MA, United States of America; Temple University School of Medicine, UNITED STATES

## Abstract

**Objectives:**

Opioids have immunosuppressive properties, yet opioid effects on T cell abnormalities consistent with the immune risk phenotype among HIV-infected individuals are understudied.

**Methods:**

To assess associations between illicit opioid use and T cell characteristics (CD4/CD8 ratio, memory profiles based on CD45RO and CD28 expression, and senescence based on CD57 expression), we conducted an exploratory cross-sectional analysis of *Russia ARCH*, a cohort of antiretroviral therapy (ART)-naïve HIV-infected individuals recruited 11/2012 to 10/2014 in St. Petersburg, Russia. The main independent variable was past 30 day illicit opioid use (yes vs. no). Secondary analyses evaluated none (0 days), intermittent (1 to 7 days), and persistent (8 to 30 days) opioid use. Outcomes were determined with flow cytometry. Analyses were conducted using linear regression models.

**Results:**

Among 186 participants, 38% reported any illicit opioid use (18% intermittent and 20% persistent). Any illicit opioid use was not significantly associated with T cell characteristics. Intermittent opioid use appeared to be associated with decreased memory CD8+ T cells proportion (CD45RO+CD45RA- CD8+ T cells: adjusted mean difference [AMD] [95% CI] = -6.15 [-11.50, -0.79], p = 0.02) and borderline significant increased senescent T cells (%CD57+ of total CD28-CD8+ T cells (AMD [95% CI] = 7.70 [-0.06, 15.46], p = 0.05).

**Conclusions:**

Among ART-naïve HIV-infected Russians, any illicit opioid use was not significantly associated with T cell abnormalities although intermittent illicit opioid use may be associated with CD8 T cell abnormalities. Longitudinal studies are warranted to confirm these findings given increased risk of infections and comorbidities seen among HIV-infected individuals with illicit opioid use.

## Introduction

Approximately 3 million people who inject drugs worldwide are HIV-infected[1]. Active opioid use is associated with adverse HIV-related behavioral and medical outcomes, including decreased access to care and linkage to treatment, antiretroviral therapy (ART) non-adherence and increased prevalence of comorbid conditions (e.g. hepatitis C virus infection)[2–4]. Furthermore, data suggest that opioids may have a range of direct effects on the immune system[5–7].

Of particular relevance for HIV-infected individuals is the potential for opioids to impact T cell levels and function, however, variable effects have been observed. *In vitro* studies indicate that opioid exposure promotes lymphocyte apoptosis and impairs T cell proliferation [8, 9], however, in the presence of HIV may prevent lymphocyte apoptosis [10]. Animal studies demonstrate that both chronic opioid exposure[11] and withdrawal[12] alter T cell dynamics. A number of epidemiologic studies have examined the effects of illicit opioids on CD4+ cell count with mixed results[2, 5, 13, 14]. Few studies, however, have focused on other important aspects of the adaptive immune system associated with the “immune risk phenotype,” including inverted CD4+/CD8+ ratio, expansion of memory T cells and evidence of senescent cells (i.e. poor replicative capacity)[15, 16]. In the elderly population, these changes have been associated with morbidity and mortality[15, 16]. Similar to the immune risk phenotype as described in the elderly, HIV-infected individuals also demonstrate inverted CD4/CD8 ratio, expansion of memory T cells, and evidence of T cell senescence[16–21]. The mechanisms driving this development of the immunosenescent phenotype are under active investigation but are believed to involve accumulation of late-stage differentiated memory T cells, with cells that have shortened telomere, lack replicative capacity, and secrete inflammatory cytokines [22]. However, the contribution of illicit opioid use to these T cell aberrancies is not currently well described.

Therefore, we conducted an exploratory analysis to assess the association between illicit opioid use (i.e. any use and patterns of use) and aspects of the immune risk phenotype in a sample of HIV-infected individuals. In order to mitigate potential confounding of antiretroviral therapy (ART) and comorbid substance use (i.e., cocaine, prescription opioids) and its associated treatment (i.e., opioid agonist treatment with methadone or buprenorphine), we conducted this study in a cohort of ART-naïve individuals living in Russia. In this setting, the confounders are rare as opioids are the main illicit drug and there is very limited availability of prescription opioids for pain and no opioid agonist therapy for opioid use disorders. Additionally, at the time of the study, ART was not widespread in the region. These Russian realities enabled a straightforward examination of the association between opioid use and immune risk phenotype among untreated HIV-infected individuals[23–26].

## Methods

### Study design

We conducted a cross-sectional analysis of data from the *Russia ARCH* cohort to explore the association between illicit opioid use and immune risk phenotype, as measured by inverted CD4+/CD8+ ratio, expansion of memory CD4+ and CD8+ T cell profiles, and senescent CD8+ T cells.

### Study participants

Participants were recruited between November 2012 and October 2014 from clinical HIV and addiction care sites, non-clinical sites and snowball recruitment to participate in an observational cohort study in St. Petersburg, Russia. This prospective study recruited individuals who met the following eligibility criteria: 1) age 18 to 70 years old; 2) HIV-infected; 3) provided information for at least two contacts; 4) had a stable address within St. Petersburg or districts within 100 kilometers of the city; 5) possessed a home or a mobile phone; and 6) were ART-naïve at the time of enrollment. Participants were excluded if they were not fluent in Russian or had cognitive impairment resulting in inability to provide informed consent.

For the current analysis, the following additional eligibility criteria were included: 1) available survey and laboratory data; 2) time since HIV diagnosis at least one year given the unique changes in the immune system during this time; and 3) detectable viral load defined as HIV viral load > 500 copies/mL on initial or repeat laboratory testing, consistent with not being on ART.

The study was approved by the institutional review boards of Boston University Medical Campus/Boston Medical Center and First St. Petersburg Pavlov State Medical University. All participants provided written informed consent and were reimbursed the equivalent of USD $33 for their completion of the baseline visit.

### Assessments

Data were collected through in-person interviews and blood collection. For this analysis, baseline data were used. All laboratory assays were performed at St. Petersburg Pasteur Institute Central Clinical Diagnostic Laboratory. Blood was collected in heparin anticoagulated tubes and processed within four hours of phlebotomy. Whole blood was stained with three independent antibody panels, each in a 100 uL volume. All antibodies were from BD Biosciences. Panel 1 included FITC anti-CD8 (#561948), PE anti-CD45RO (#555493), PeCy5 anti-CD45RA (#555490) and APC-H7 anti-CD4 (#5601058). Panel 2 included FITC anti-CD8 (#561948), PE anti-CD57 (#560844), PeCy5 anti-CD28 (#555730) and APC-H7 anti CD4 (#560158). Panel 3 included appropriate isotype controls. Samples were incubated at room temperature in the dark for 20 minutes, red blood cells were lysed using BD Pharmlyse, and the cells were washed and fixed in paraformaldehyde in preparation for analysis on flow cytometer. Flow cytometry was performed on a BD FACS Canto, using single color controls for machine compensation. Data was analyzed with BD FACS DIVA software. Lymphocytes were gated based on forward and side scatter, and %CD4 and %CD8 were based on gated lymphocytes. Memory and senescent markers were gated on %CD4 and %CD8 unless otherwise indicated.

### Outcomes

We defined three T cell abnormalities consistent with the immune risk phenotypes as the outcomes of interest: 1) inverted CD4+/CD8+ ratio, 2) expansion of CD4+ and CD8+ memory T cells, and 3) increased CD8+ T cell senescence (**Table 1**). Specifically, to determine expansion of CD4+ and CD8+ memory T cells, we examined the proportion of memory CD4+ and CD8+ T cells (%CD45RO+CD45RA- of total CD4+ and %CD45RO+CD45RA- of total CD8+). We then examined the proportion of CD8+ T cells with loss of CD28 expression (i.e. %CD28- of total CD8+). Both CD45RO and loss of CD28 expression have been used previously to define memory T cells[15, 27, 28]. To examine increased CD8+ T cell senescence, we examined the proportion of CD28+ and CD28- T cells with CD57 expression (i.e. %CD57+ of CD28+CD8+ and %CD57+ of CD28-CD8+, respectively) and the proportion of CD28-CD57+ of total CD8+ T cells (i.e. %CD28-CD57+ of CD8+). CD57 is a marker of T cell senescence, and is associated with poor capacity to replicate[15, 29]. While the understanding of the significance of these markers has continued to evolve, these markers were chosen based on the current information available at the initiation of this study to identify markers of memory and immune senescence. All outcomes were analyzed as continuous variables.

**Table 1:** Outcomes of interest: T cell abnormalities consistent with immune risk phenotype.

Outcome of Interest	Description
Inversion of CD4+/CD8+ ratio	Normal ratio: 2 to 1. A ratio less than 1 occurs with HIV infection and other inflammatory conditions and aging. Inversion of the ratio occurs due to drops in CD4+ cells and/or expansion of CD8+ cells.
Expansion of CD4 and CD8+ memory cells: • %CD45RO+CD45RA-CD4+ of total CD4+ • %CD45RO+CD45RA-CD8+ of total CD8+ • %CD28- of total CD8+	As measured by CD45RO expression/loss of CD45RA and loss of CD28 expression, a co-receptor required for T cell activation, expansion of memory T cells and reflect persistent antigen exposure.
Increased CD8+ T cell senescence: • %CD57+ of total CD28+CD8+ • %CD57+ of total CD28-CD8+ • % CD57+CD28- of total CD8+	As measured by gain of CD57+ expression, these T cells have decreased replicative capacity.

### Main independent variable: Illicit opioid use

Self-reported substance use was assessed using a modified Risk Behaviors Survey (RBS)[30–32]. The main independent variable was illicit opioid use (none vs. any), based on whether participants reported any use of heroin or other opioids in the past 30 days. In secondary analyses, we also examined the effects of pattern of illicit opioid use. Pattern of use was a 3-level variable defined by self-reported number of days of use of heroin or other opioids in the past 30 days and participants were categorized as having none (0 days), intermittent (1 to 7 days) and persistent (8 to 30 days) illicit opioid use.

### Covariates

Potential confounders were selected based on the literature and clinical knowledge. Socio-demographic characteristics included age and gender. HIV disease characteristics included HIV viral load and self-reported time since diagnosis. Depressive symptoms were evaluated using the Center for Epidemiologic Studies Depression Scale-D (CES-D) defined as a score greater than or equal to 16[33–35]. Prior history of infections, including hepatitis C, hepatitis B, tuberculosis and shingles, were based on self-report[36]. Participants were considered to be regular smokers if they reported use of at least one cigarette per day or an average of at least 7 cigarettes per week[37, 38]. Past 30 day presence of heavy alcohol use, ascertained with the interview-based 30-day Time-Line Follow Back (TLFB)[39], was defined as alcohol use exceeding the National Institute on Alcohol Abuse and Alcoholism guidelines (i.e. more than 14 drinks per week or 5 drinks per occasion for men less than or equal to 65 years old and more than 7 drinks per week or 4 drinks per occasion for all women and men over 65 years old)[40]. Past 30 day stimulant (typically ephedrine and/or crack or cocaine in Russia) and cannabis use, assessed with a modified RBS[30, 31], were also analyzed as dichotomous variables.

### Statistical analysis

We used descriptive statistics to characterize the demographic and clinical characteristics of the analytic sample, overall and stratified by illicit opioid use, and compared groups using chi-square and Fisher’s exact tests for categorical variables and t-tests and Wilcoxon tests for continuous variables, as appropriate. We estimated the proportion with each pattern of illicit opioid use (i.e. none, intermittent, and persistent). Correlations between independent variables and covariates was assessed using Spearman correlation and no pair of variables included in the regression models had correlation >0.40. Due to moderate correlation between stimulant and cannabis use (r = 0.47), only stimulant use was included in the regression models. In these exploratory analyses, separate multiple linear regression models were constructed to estimate the unadjusted and adjusted associations between presence of illicit opioid use and each outcome of interest: inverted CD4+/CD8+ ratio, expansion of memory CD4+ and CD8+ T cell subsets, and increased CD8+ T cell senescence. We conducted a series of adjusted analyses to control for potential confounding factors. The first model included age, gender, log_10_ HIV viral load, time since HIV diagnosis and depressive symptoms. The second set of models additionally included co-infections (i.e. hepatitis C, hepatitis B, tuberculosis, and shingles). The third set of models included other substance use in addition to the other covariates. The third set of models, fully adjusted for all potential confounders, was considered the final analysis. In secondary analyses, we examined the effects of pattern of illicit opioid use on each of our outcomes of interest using the same approach described above. We report the adjusted mean differences (AMD) and the associated 95% confidence intervals (95% CI) corresponding to the main independent variables. In confirmatory analyses, we refit the final models using median regression models[41, 42] as this method does not rely on the normality assumption and is more robust to outliers than linear regression. Due to the exploratory, hypothesis-generating nature of these analyses, no adjustments were made for multiple comparisons. Two-tailed tests and an alpha level of 0.05 were used for all tests. All analyses were performed using SAS version 9.3 (SAS Institute, Inc, NC, USA).

## Results

### Participant characteristics

Among the 253 *Russia ARCH* participants with available flow cytometry data, 30 were excluded due to an HIV diagnosis within 1 year and 37 due to an undetectable viral load. Among the 186 participants included in the final analytic sample, 38% reported past 30-day illicit opioid use (**Table 2**). Participants were a mean age of 33 years old, 73% male, had been diagnosed with HIV for a mean of 8 years, and 86% reported being exposed to HIV through injection drug use. Depressive symptoms were common among those without and with illicit opioid use (43% vs. 56%, p = 0.07). Compared to those without illicit opioid use, hepatitis C (87% vs. 97%, p = 0.02) and hepatitis B (34% vs. 58%, p = 0.001) were more common among those with illicit opioid use, while tuberculosis (5% vs. 13%, p = 0.07) and shingles (22% vs. 30%, p = 0.23) were similarly experienced. Compared to those without illicit opioid use, those with illicit opioid use were more likely to be regular smokers (78% vs. 96%, p = 0.001) and report stimulant (6% vs. 17%, p = 0.02) and cannabis use (11% vs.25%, p = 0.01). Heavy alcohol use was common in both groups (without and with illicit opioid use: 51% vs. 66%, p = 0.05).

**Table 2:** Participant demographic and clinical characteristics.

Characteristic	Overall, n = 186	Past 30 Day Illicit Opioid Use: No, n = 115 (61.8%)	Past 30 Day Illicit Opioid Use: Yes, n = 71 (38.2%)	p value
**Demographics**				
Age, mean (SD)	33.1 (4.8)	33.2 (5.0)	33.0 (4.5)	0.76
Gender, male, n (%)	136 (73.1%)	85 (73.9%)	51 (71.8%)	0.76
**HIV Disease Characteristics**				
Log_10_ HIV viral load, mean (SD)	4.6 (0.9)	4.5 (0.9)	4.7 (1.0)	0.10
Time since HIV diagnosis, years, mean (SD)	8.1 (4.1)	8.2 (4.4)	8.0 (3.7)	0.66
HIV risk category, n (%)				0.39
IDU	157 (85.8%)	92 (82.1%)	65 (91.6%)	
MSM	3 (1.6%)	3 (2.7%)	0 (0.0%)	
MSM/IDU	3 (1.6%)	2 (1.8%)	1 (1.4%)	
Heterosexual sexual contact	19 (10.4%)	14 (12.5%)	5 (7.0%)	
Perceived heterosexual/ Received blood products	1 (<1%)	1 (0.9%)	0 (0.0%)	
**Symptoms and Comorbid Infections**				
Depressive symptoms, n (%)	88 (47.8%)	48 (42.5%)	40 (56.3%)	0.07
Hepatitis C, n (%)	169 (90.9%)	100 (87.0%)	69 (97.2%)	**0.02**
Hepatitis B, n (%)	80 (43.0%)	39 (33.9%)	41 (57.7%)	**0.001**
Tuberculosis, n (%)	15 (8.1%)	6 (5.2%)	9 (12.7%)	0.07
Shingles, n (%)	46 (24.7%)	25 (21.7%)	21 (29.6%)	0.23
**Substance Use**				
Regular smoker, n (%)	158 (84.9%)	90 (78.3%)	68 (95.8%)	**0.001**
Heavy alcohol use, n (%)	106 (57.0%)	59 (51.3%)	47 (66.2%)	0.05
Stimulant use, n (%)	19 (10.2%)	7 (6.1%)	12 (16.9%)	**0.02**
Cannabis use, n (%)	31 (16.7%)	13 (11.3%)	18 (25.4%)	**0.01**
**T cell subsets**				
CD4/CD8 ratio, mean (SD)	0.78 (0.49)	0.83 (0.51)	0.71 (0.44)	0.09
%CD45RO+CD45RA-CD4+ of total CD4+, mean (SD)	53.02 (17.12)	52.70 (16.74)	53.54 (17.82)	0.75
%CD45RO+CD45RA-CD8+ of total CD8+, mean (SD)	34.00 (13.69)	33.94 (12.99)	34.10 (14.84)	0.94
%CD28- of total CD8+, mean (SD)	61.16 (12.48)	60.41 (12.24)	62.37 (12.87)	0.30
%CD57+ of total CD28+CD8+, mean (SD)	17.56 (21.04)	15.39 (18.52)	21.09 (24.32)	0.07
%CD57+ of total CD28-CD8+, mean (SD)	56.54 (18.67)	54.76 (19.09)	59.42 (17.73)	0.10
% CD57+CD28- of total CD8+, mean (SD)	34.79 (14.33)	33.38 (14.52)	37.08 (13.82)	0.09

**Notes:** bold text indicates statistical significant at p<0.05 level.

SD = standard deviation. IDU = injection drug use; MSM = men who have sex with men.

### Illicit opioid use

No illicit opioid use was reported by 62% of participants, while 18% reported intermittent use and 20% reported persistent use. Among those reporting illicit opioid use, the mean number of days of use over the past 30 days was 12 (SD 11). Regarding type of illicit opioid use in the past 30 days, 27% reported heroin use by itself or with stimulants; 39% reported opioid use other than heroin without other drugs and 34% reported heroin in combination with other opioids.

### Illicit opioid use and CD4+/CD8+ ratio and memory T cell subsets

There were no differences in observed measures of T cell subsets based on illicit opioid use (all p values>0.05) (**Table 2**). In unadjusted and all adjusted models, any illicit opioid use compared to none was not significantly associated with any of the measured outcomes: inverted CD4+/CD8+ ratio or expansion of memory T cells (as measured by the proportion of CD45RO+CD45RA- CD4+ or CD8+ T cells and proportion of CD28- of total CD8+ T cells) (**Table 3**). In secondary analyses, we found that compared to no illicit opioid use, intermittent use was associated with a significant decrease in the proportion of memory CD8+ T cells as measured by CD45RO+CD45RA- CD8+ T cells (AMD [95% CI] = -6.15 [-11.50, -0.79], p = 0.02) in final adjusted models (**Table 4**). In contrast, compared to no opioid use, there was no significant effect of persistent opioid use on this subset of T cells (AMD [95% CI] = 3.96 [-1.34, 9.26], p = 0.14). Pattern of opioid use was not significantly associated with any other outcomes of interest. In confirmatory analyses using median regression, the results were consistent for each of these outcomes.

**Table 3:** Association between illicit opioid use and CD4/CD8 ratio and memory cells, linear regression models.

	CD4/CD8 Ratio	%CD45RO+CD45RA-CD4+ of total CD4+	%CD45RO+CD45RA-CD8+ of total CD8+	%CD28- of total CD8+
	Adjusted mean difference (95% Confidence Interval)			
**Unadjusted**	-0.12 (-0.27, 0.02)	0.84 (-4.27, 5.95)	0.16 (-3.93, 4.24)	1.96 (-1.75, 5.68)
**Adjusted Model**^**a**^	-0.08 (-0.22, 0.06)	0.50 (-4.62, 5.61)	-0.20 (-4.25, 3.85)	1.21 (-2.48, 4.91)
**Adjusted Model**^**a**^ **+ comorbid infections**^**b**^	-0.05 (-0.20, 0.09)	-0.39 (-5.70, 4.91)	-0.90 (-5.15, 3.35)	2.02 (-1.85, 5.89)
**Final Adjusted Model**^**a**^^,^^**b**^ **+ Other Substance Use**^**c**^	-0.04 (-0.19, 0.11)	-1.15 (-6.62, 4.32)	-1.02 (-5.40, 3.35)	1.61 (-2.37, 5.59)

Notes

A. Model includes gender, age, log HIV viral load, time since HIV diagnosis, and depressive symptoms.

B. Comorbid infections includes hepatitis C, hepatitis B, tuberculosis and shingles.

C. Other substance use includes regular smoker, heavy alcohol use and stimulant use.

**Table 4:** Association between pattern of illicit opioid use and CD4/CD8 ratio and memory cells, linear regression models*.

	CD4/CD8 Ratio	%CD45RO+CD45RA-CD4+ of total CD4+	%CD45RO+CD45RA-CD8+ of total CD8+	%CD28- of total CD8+
	Adjusted mean difference (95% Confidence Interval)			
**Intermittent Use vs. None**	0.01 (-0.18, 0.20)	-3.66 (-10.51, 3.19)	**-6.15 (-11.50, -0.79)**	-0.18 (-5.17, 4.82)
**Persistent Use vs. None**	-0.08 (-0.27, 0.10)	1.29 (-5.49, 8.08)	3.96 (-1.34, 9.26)	3.35 (-1.60, 8.29)

Notes

*Models adjusted for gender, age, log HIV viral load, time since HIV diagnosis, depressive symptoms, comorbid conditions (hepatitis C, hepatitis B, tuberculosis, and shingles), and other substance use (regular smoker, heavy alcohol use and stimulant use). Bold text indicates p<0.05.

### Illicit opioid use and T cell senescence

In unadjusted and adjusted models, any illicit opioid use compared to none was not significantly associated with an increase in CD8+ T cell senescence (as measured by CD57 expression) (**Table 5**). In secondary analyses, we found that compared to no illicit opioid use, intermittent opioid use was associated with a borderline significant increase in %CD57+ of CD28-CD8+ T cells (AMD [95% CI] = 7.70 [-0.06, 15.46], p = 0.05) in final adjusted models (**Table 6**). In contrast, compared to no use, levels of CD57 expression were not significantly different for those with persistent opioid use (AMD [95% CI] = 2.48 [-5.21, 10.16], p = 0.53). There were no other significant associations between pattern of opioid use and CD57 expression among any of the other CD8 T cell subsets (all p values>0.05). Results were consistent in confirmatory analyses using median regression models.

**Table 5:** Association between illicit opioid use and CD8 T cell senescence, linear regression models.

	%CD57+ of total CD28+CD8+	%CD57+ of total CD28-CD8+	% CD57+CD28- of total CD8+
	Adjusted Mean Difference (95% Confidence Interval)		
**Unadjusted**	5.70 (-0.53, 11.93)	4.66 (-0.87, 10.20)	3.70 (-0.54, 7.95)
**Adjusted Model**^**a**^	4.98 (-1.37, 11.33)	4.32 (-1.38, 10.02)	3.06 (-1.28, 7.41)
**Adjusted Model**^**a**^ **+ Comorbid Infections**^**b**^	4.52 (-2.14, 11.17)	4.79 (-1.21, 10.80)	3.93 (-0.66, 8.52)
**Final Adjusted Model**^**a**^^**,**^^**b**^ **+ Other Substance Use**^**c**^	3.78 (-3.10, 10.66)	5.06 (-1.13, 11.24)	3.83 (-0.86, 8.51)

Notes

A. Model includes gender, age, log HIV viral load, time since HIV diagnosis, and depressive symptoms.

B. Comorbid infections includes hepatitis C, hepatitis B, tuberculosis and shingles.

C. Other substance use includes regular smoker, heavy alcohol use and stimulant use.

**Table 6:** Association between pattern of illicit opioid use and CD8 T cell senescence, linear regression models*.

	%CD57+ of total CD28+CD8+	%CD57+ of total CD28-CD8+	% CD57+CD28- of total CD8+
	Adjusted Mean Difference (95% Confidence Interval)		
**Intermittent Use vs. None**	3.51 (-5.16, 12.17)	7.70 (-0.06, 15.46)	4.00 (-1.90, 9.89)
**Persistent Use vs. None**	4.05 (-4.53, 12.63)	2.48 (-5.21, 10.16)	3.66 (-2.18, 9.50)

Notes

*Models adjusted for gender, age, log HIV viral load, time since HIV diagnosis, depressive symptoms, comorbid conditions (hepatitis C, hepatitis B, tuberculosis, and shingles), and other substance use (regular smoker, heavy alcohol use and stimulant use). Bold text indicates p<0.05.

## Discussion

In these exploratory analyses among a sample of young, HIV-infected, ART-naïve Russians, we found that any illicit opioid use was generally not significantly associated with measures of T cell abnormalities consistent with the immune risk phenotype. In secondary analyses, we found that intermittent illicit opioid use appeared to be associated with a decrease in the proportion of memory CD8 T cells and a borderline significant increase in CD57 expression among CD28-CD8+ T cells. We did not detect a significant association between illicit opioid use and an inverted CD4+/CD8+ ratio, or memory T cells (based on CD45RO+CD45RA- or loss of CD28). Taken together, these data indicate that these T cell abnormalities are unlikely to explain the negative health outcomes, including comorbid conditions, observed among those with illicit opioid use in the setting of untreated HIV infection.

Previous literature indicates that opioids directly modulate T cell dynamics and function. For example, data from rhesus macaques reveal that chronic morphine exposure is associated with increased levels of regulatory T (Treg) cells, increased activity of Th17 cells, and altered surface expression of T cells (i.e. CD161, CCR6, CCR5 and β7 integrin)[11]. Moreover, studies in humans have demonstrated that active heroin use is associated with impaired lymphocyte proliferation and altered T cell subsets (i.e. Th1/Th2, Tregs), which is restored with opioid agonist therapy[14, 43]. These prior studies, however, have focused on relatively small sample sizes and have been done in the general population[14, 43–45]. To our knowledge, no published studies have specifically examined the association between opioid use and alterations in T cell subsets among HIV-infected patients.

Studies indicate that older age and chronic viral infections are associated with increased CD57 expression. CD57 expression, which is virtually non-existent at birth, increases over the life span and is attributed to accumulative exposure to chronic viral infections[15]. Predictive of poor cellular replicative capacity, CD57 expression on CD8+ T cells, is associated with morbidity (e.g., malignancy) and mortality. Among HIV-infected individuals, increased CD57 expression has been associated with CMV-seropositive status[46] and negative outcomes in some studies, including subclinical atherosclerosis[47], Kaposi’s sarcoma[48], and mortality[20]. In contrast, another study found that decreased CD57 expression among CD28-CD8+ T cells was associated with mortality risk in a sample of virally suppressed HIV-infected individuals, who had low nadir CD4 counts[49]. Notably, the observed CD57 expression in this prior study was lower than that identified in the current study, likely relating to differences in duration of HIV infection and use of ART. Our findings in this cohort of ART-naïve HIV-infected individuals raise the possibility that like aging, intermittent illicit opioid use may promote T cell differentiation, proliferation and subsequent immunosenescence. The hypothesis raised by these findings is that intermittent illicit opioid use may amplify such risk among HIV-infected patients; such a hypothesis is consistent with data from the general population indicating that opioid use is associated with increased cardiovascular disease and mortality[50–52].

Our findings suggest that, in comparison to no use and persistent use, intermittent opioid use may have differential effects on the immune system[12, 13, 45, 53]. We found that, compared to no opioid use, intermittent illicit opioid use was associated with a borderline significant increase in %CD57+ of CD28-CD8+ T cells, and a decrease in memory CD45RO+CD45RA- CD8+ T cells. Why intermittent opioid use appears to be associated with more T cell senescence is of interest and deserves further exploration. These findings may be explained by the previously recognized harmful effects associated with opioid withdrawal[12] on certain aspects of the immune system. In addition, these results are consistent with our previous pilot study which found that among a sample of young, HIV-infected ART-naïve Russians with risky alcohol use, intermittent heroin use appeared to be associated with larger decreases in CD4 cell counts over a 12 month period, compared to no heroin use; those with persistent heroin use appeared to have an increase in CD4 cell count over time[13].

That we did not detect a significant association between any illicit opioid use and the CD4+/CD8+ ratio, CD45RO+CD45RA- CD4+ or CD8+ T cells or loss of CD28 expression among CD8+ T cells may be due to several reasons. First, effects of untreated HIV infection, which are associated with profound alterations in T cells, including a lower CD4+/CD8+ ratio [21, 54, 55], loss of CD28 expression[19], and T cell senescence, may mask more subtle effects associated with illicit opioid use. Second, it is possible that illicit opioid use is selectively associated with the development of impaired replicative capacity, as manifested by CD57+ expression, without impacting other aspects of CD8+ T cells. Third, given that this study was conducted in a relatively young cohort, it may be that their immune systems are more resilient to effects of illicit opioids that might be apparent in an older cohort.

Our study should be interpreted in the context of its limitations. First, our study did not include staining for CD3, a marker to define T cells. We are unable to define how much other white blood cells (e.g. NK cells) were captured in these analyses. Future studies should include CD3 or other markers to define T cells adding specificity to this line of research. Second, this is a cross-sectional study, we were unable to make any conclusions about causality. Third, we relied on self-reported history of illicit opioid use and other important covariates (e.g. hepatitis C and hepatitis B), which may be under reported. In addition, we focused on recent (i.e. past 30 day) opioid use. While the majority of participants had a history of injection drug use, we are unable to examine the effects of duration of opioid use as we did not collect data on lifetime use. In addition, we were unable to precisely quantify opioid “dose” given variability of opioid preparations. However, our categorization of none, intermittent, and persistent was a step towards examining “dose-response” effects of opioid use with the available data. Fourth, as this study was conducted in a younger population, our findings may not be generalizable to older HIV-infected individuals. Fifth, since CD45RA+ is expressed on both naïve and terminally differentiated T cells, relying on CD45RO+CD45RA- to define memory cells may under-estimate the true levels of memory cells[28]. This does not, however, diminish our finding of decreased CD45RO+CD45RA- CD8+ cells with intermittent opioid use. In addition, because CD45RA/CD45RO and CD28 were not measured in the same panel, ability to further delineate central memory and effector memory cells was not possible. These markers were utilized at the inception of this study based on current knowledge and availability, but since that time, progress in this realm has yielded characterization and understanding of T cell memory and senescent subsets that now includes multiple markers. Future studies should utilize additional markers of T cell differentiation which would allow us to ascertain whether particular T cell subsets are differentially impacted by illicit opioid use. Sixth, we did not have measures of CMV seropositivity, which have been previously demonstrated to be associated with T cell abnormalities and the immune risk phenotype. Lastly, we did not measure all T cell subtypes (e.g. regulatory T cells) or assess T cell function or markers of proliferation, which may be differentially impacted by illicit opioid use. These limitations notwithstanding, this study serves to extend the existing literature by examining the effects of illicit opioid use in a unique population, where issues related to confounding are minimized given the prevalence of different types of illicit opioid use; limited availability of prescription opioids for pain; lack of opioid agonist therapy; and restricted use of ART.

In conclusion, our study indicates that illicit opioid use generally does not appear to be associated with markers of the immune risk phenotype in a younger cohort of HIV-infected individuals not on ART. To confirm these findings, future studies with larger sample sizes examining the longitudinal associations between illicit opioid use and CD8+ T cell abnormalities among treated and untreated HIV-infected, and including uninfected patients with and without illicit opioid use, are indicated. Furthermore, the finding that intermittent opioid use may be associated with increasing T cell senescence requires further exploration in other cohorts of HIV-infected individuals, using detailed immunophenotyping, among individuals both on and off ART. In addition, future studies examining the impact of opioid use, as well as the potential role of particular opioid receptors (i.e. mu, kappa, delta), on T cell function are warranted. Observations that illicit opioid use is associated with increased infectious complications and comorbidities do not appear to be explained by abnormalities in memory or senescence T cells in a cohort of younger HIV-infected individuals.

## Supporting information

S1 File PONE_illicit_opioid_use. (CSV)
